# 312. Chlorhexidine Gluconate (CHG) Skin Concentration Measurement and Feedback in Hematology-Oncology and Hematopoietic Stem Cell Transplant (HO/HSCT) Units: A Multicenter Study

**DOI:** 10.1093/ofid/ofaf695.108

**Published:** 2026-01-11

**Authors:** Yoona Rhee, Lahari Thotapalli, Michael Schoeny, Ahmed Babiker, Erik Dubberke, Scott Fridkin, Erin Gettler, Surbhi Leekha, David K Warren, Matthew J Ziegler, Rachel Addison, Katherine Foy, Mary Carl Froilan, Tracey Habrock-Bach, Elizabeth C Huang, McKenzi King, Michelle Newman, Katheryn Ney, Deepti Suchindran, Pam C Tolomeo, Jae Jung, Maitri Shah, Ellen Gough, Mary K Hayden, Michael Y Lin

**Affiliations:** Rush University Medical Center, Chicago, IL; Rush University Medical Center, Chicago, IL; Rush University College of Nursing, Chicago, Illinois; Rush University Medical Center, Chicago, IL; Washington University School of Medicine; Georgia Emerging Infections Program, Decatur, GA; Emory University School of Medicine, Atlanta, GA, Atlanta, Georgia; Duke University Hospital, Durham, NC; University of Maryland School of Medicine, Baltimore, MD; University of Nebraska Medical Center, Omaha, Nebraska; University of Pennsylvania, Philadelphia, Pennsylvania; Duke Center for Antimicrobial Stewardship and Infection Prevention, Durham, NC; Duke University, Durham, North Carolina; Rush University Medical Center, Chicago, IL; Washington University School of Medicine; University of Pennsylvania CCEB, Philadelphia, Pennsylvania; Rush University Medical Center, Chicago, IL; University of Maryland Baltimore, Baltimore, Maryland; Washington University School of Medicine; Emory University, Atlanta, Georgia; University of Pennsylvania, Philadelphia, Pennsylvania; Rush University Medical Center, Chicago, IL; Rush University Medical Center, Chicago, IL; Rush University Medical Center, Chicago, IL; Rush University Medical Center, Chicago, IL; Rush University Medical Center, Chicago, IL

## Abstract

**Background:**

The quality of CHG bathing for infection prevention in HO/HSCT units is uncertain, as patients are often self-bathing. We assessed CHG bathing quality by measuring patients’ CHG skin concentrations at baseline and then evaluated whether feedback of results to unit leadership/staff could guide improvement.

**Table.**
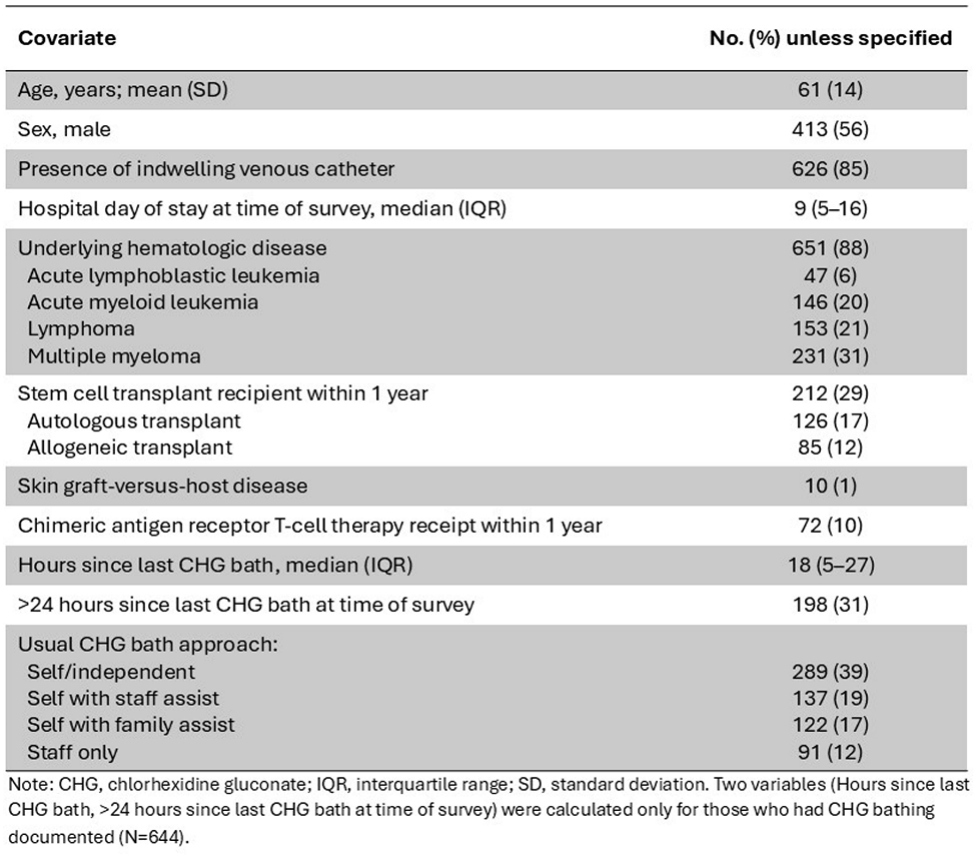
Demographics and Clinical Factors for Patients in Hematology-Oncology/Hematopoietic Stem Cell Transplant Units from Six Hospitals (N=736)

**Figure 1.**
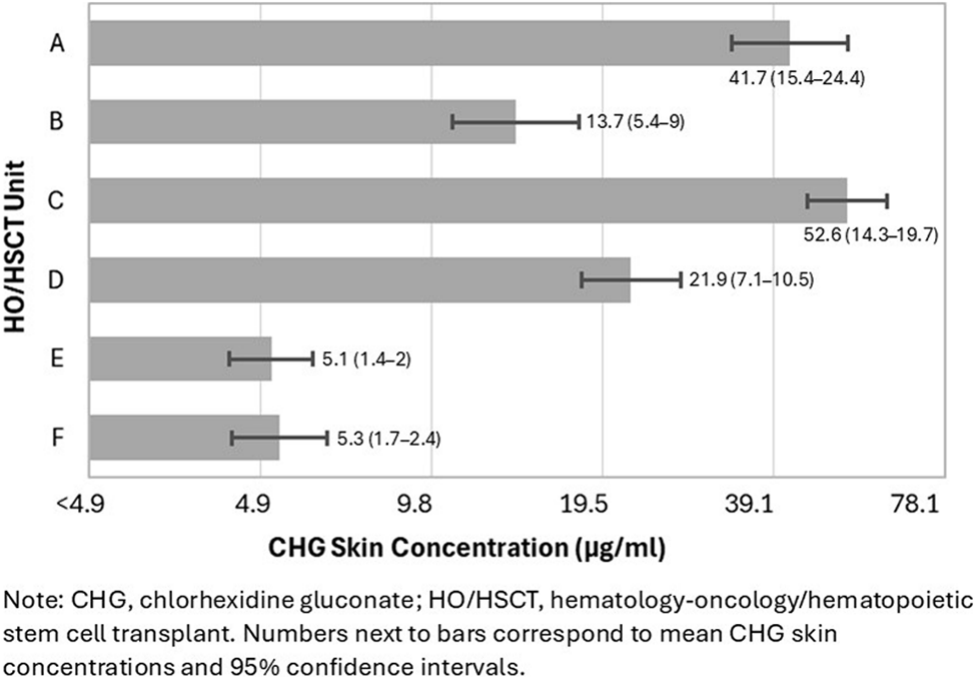
Baseline Chlorhexidine Gluconate Skin Concentrations in Six Hematology-Oncology/Hematopoietic Stem Cell Transplant Units (N=379)

**Methods:**

We conducted 6 point prevalence surveys from 9/2023 – 10/2024 in HO/HSCT units that performed routine CHG bathing at 6 hospitals. During each survey, we collected swab samples of patients’ skin (neck, axilla, inguinal region) and clinical data. We used a colorimetric assay to measure CHG skin concentrations (detection range ≥4.9 to 20,000 µg/ml). During surveys 1-3 (baseline period), CHG concentrations were measured but not shared. During surveys 4-6 (intervention period), summary CHG concentrations from baseline and after each intervention period survey were shared with unit leadership/staff, who led local quality improvement initiatives. We used linear and logistic regression to model outcomes, controlling for clustering as appropriate.

**Figure 2.**
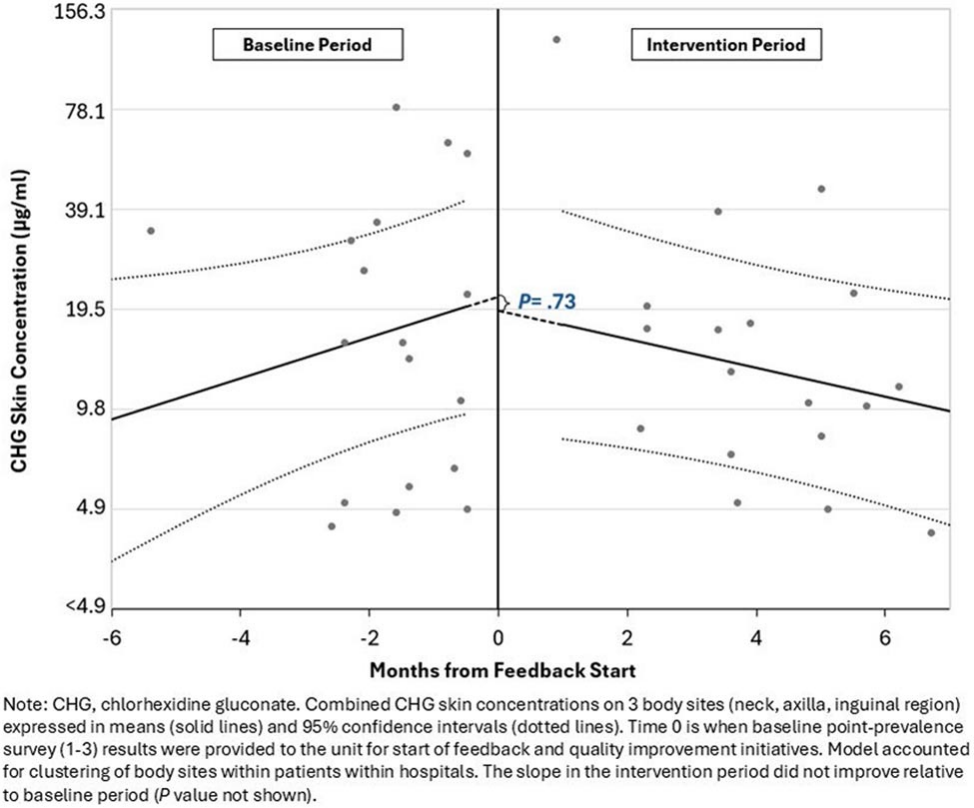
Modeled Chlorhexidine Gluconate Skin Concentrations between Baseline and Intervention Periods in Hematology-Oncology/Hematopoietic Stem Cell Transplant Units from Six Hospitals (N=736)

**Results:**

Six units participated (median 34 beds/unit); 3 units only used 2% CHG-impregnated cloth, and 3 units used bathing approaches that included other CHG formulations (4% CHG liquid or foam). Of 949 eligible patients, 83% consented to participate; after deduplication, 736 unique patients were analyzed (Table). 87% of patients reported using CHG. At baseline, 22% of 379 patients had undetectable CHG skin concentrations on all 3 body sites; CHG concentrations varied by unit (*P*< .001, Figure 1). In adjusted analyses, there was no difference between baseline and intervention periods in CHG skin concentration levels (*P*=.73; Figure 2) or CHG detection on any body site (*P*=.40). CHG-impregnated cloth-exclusive units had 4-fold higher CHG skin concentrations than non-cloth exclusive units (*P*=.005). Patients who had CHG baths performed by staff only or with staff assistance had 31% higher CHG skin concentrations than those without staff involvement (*P*=.02).

**Conclusion:**

We found significant variation in CHG bathing quality among HO/HSCT patients and between units; unit-level feedback of CHG measurements did not lead to improvement. We identified CHG formulation and staff assistance with CHG bathing as potential modifiable factors.

**Disclosures:**

Erik Dubberke, MD, MSPH, AstraZenca: Advisor/Consultant|AstraZenca: Grant/Research Support|Pfizer, Inc.: Advisor/Consultant|Pfizer, Inc.: Grant/Research Support|Theriva Biologics: Grant/Research Support|Vedanta Biosciences, Inc.: Advisor/Consultant|Vedanta Biosciences, Inc.: Grant/Research Support

